# Gluconeogenesis using glycerol as a substrate in bloodstream-form *Trypanosoma brucei*

**DOI:** 10.1371/journal.ppat.1007475

**Published:** 2018-12-27

**Authors:** Julie Kovářová, Rupa Nagar, Joana Faria, Michael A. J. Ferguson, Michael P. Barrett, David Horn

**Affiliations:** 1 The Wellcome Centre for Anti-Infectives Research, School of Life Sciences, University of Dundee, Dow Street, Dundee, United Kingdom; 2 The Wellcome Centre for Molecular Parasitology, Institute of Infection, Immunity and Inflammation, University of Glasgow, Glasgow, United Kingdom; 3 Glasgow Polyomics, University of Glasgow, Glasgow, United Kingdom; Biology Centre, Academy of Sciences of the Czech Republic, CZECH REPUBLIC

## Abstract

Bloodstream form African trypanosomes are thought to rely exclusively upon glycolysis, using glucose as a substrate, for ATP production. Indeed, the pathway has long been considered a potential therapeutic target to tackle the devastating and neglected tropical diseases caused by these parasites. However, plasma membrane glucose and glycerol transporters are both expressed by trypanosomes and these parasites can infiltrate tissues that contain glycerol. Here, we show that bloodstream form trypanosomes can use glycerol for gluconeogenesis and for ATP production, particularly when deprived of glucose following hexose transporter depletion. We demonstrate that *Trypanosoma brucei* hexose transporters 1 and 2 (THT1 and THT2) are localized to the plasma membrane and that knockdown of THT1 expression leads to a growth defect that is more severe when THT2 is also knocked down. These data are consistent with THT1 and THT2 being the primary routes of glucose supply for the production of ATP by glycolysis. However, supplementation of the growth medium with glycerol substantially rescued the growth defect caused by THT1 and THT2 knockdown. Metabolomic analyses with heavy-isotope labelled glycerol demonstrated that trypanosomes take up glycerol and use it to synthesize intermediates of gluconeogenesis, including fructose 1,6-bisphosphate and hexose 6-phosphates, which feed the pentose phosphate pathway and variant surface glycoprotein biosynthesis. We used Cas9-mediated gene knockout to demonstrate a gluconeogenesis-specific, but fructose-1,6-bisphosphatase (Tb927.9.8720)-independent activity, converting fructose 1,6-bisphosphate into fructose 6-phosphate. In addition, we observed increased flux through the tricarboxylic acid cycle and the succinate shunt. Thus, contrary to prior thinking, gluconeogenesis can operate in bloodstream form *T*. *brucei*. This pathway, using glycerol as a physiological substrate, may be required in mammalian host tissues.

## Author summary

Trypanosomes are the etiological agents of human sleeping sickness and animal African trypanosomiases, a range of diseases in cattle, other livestock and horses caused by several *Trypanosoma* subspecies. The mammalian stage of the parasite circulates in the bloodstream, a nutrient-rich environment with constant temperature and pH and high glucose concentration. Hence, it was unsurprising that bloodstream trypanosomes use glucose in a low-efficiency manner and produce ATP mostly from glycolysis, with simplified mitochondria and metabolism. Recently though, *T*. *brucei* were found in abundance in adipose tissue, and also in skin, suggesting the need for flexible and more elaborate metabolic capacity. We show that trypanosomes synthesise sugars *de novo* from glycerol via gluconeogenesis. Depletion of glucose transporters is rescued by supplementation with glycerol. Moreover, even wild-type parasites, grown in the presence of glucose and glycerol, use both substrates and have active gluconeogenesis. Metabolome analysis also showed utilization of glycerol to feed the pentose phosphate pathway, nucleotide biosynthesis and glycerophospholipid biosynthesis. Trypanosomes do not accumulate storage polysaccharides, but mammalian-infective parasites do assemble a dense surface glycoprotein coat, the glycan components of which incorporate carbons from glycerol. Thus, gluconeogenesis can be used to drive intermediate metabolism and terminal metabolite biosynthesis. Our results reveal metabolic flexibility and adaptability in trypanosomes, which is likely required for survival in multiple host tissue environments. This should be considered when devising metabolically targeted therapies.

## Introduction

*T*. *brucei* is the causative agent of human and animal African trypanosomiases, devastating but neglected tropical diseases. The mammalian-infective form of the parasite, typically referred to as the bloodstream form (BSF), lives in blood of mammalian hosts and enters the central nervous system (CNS), leading to a fatal disease if not treated. In addition, trypanosomes were recently detected in adipose tissue in a mouse model [1] and the skin of both humans [2] and mice [3]. Tsetse flies transmit the parasites; these procyclic forms (PCF) grow in the insect mid-gut, differentiating through other adaptive life-cycle stages, and later migrating to the salivary glands, for transmission in saliva as metacyclic forms. Each of the parasite’s stages is morphologically and metabolically adapted to the respective environmental conditions. Nutrient availability is variable in the tsetse mid-gut and PCF trypanosomes can utilize proline, and generate the majority of their ATP in a reticulated mitochondrion containing canonical functions; although the tricarboxylic acid (TCA) cycle appears to operate in a non-canonical manner [4]. On the other hand, BSF trypanosomes in the bloodstream grow in a stable, nutrient rich environment, with a constant and abundant glucose supply, producing ATP from glycolysis; the mitochondrion, the electron transport chain and the TCA cycle are substantially reduced in BSF cells [5]. The recent identification of trypanosomes in adipose tissue [1] and in the skin of humans [2] and mice [3], indicated that the ‘bloodstream forms’ should now be considered as bloodstream-resident, CNS-resident, adipose-resident or skin-resident forms, potentially with differing metabolic capacities.

Glycolysis is the metabolic pathway with the highest flux in BSF *T*. *brucei* grown in culture medium; this pathway has been thoroughly studied and has long been considered a promising potential drug target [6]. The majority of the glycolytic enzymes are localized inside glycosomes, specialized peroxisomes harboring glycolysis and additional metabolic pathways [7]. The glycosome membrane is semi-permeable; hence only smaller metabolites can pass freely [8], while ADP/ATP or NAD^+^/NADH regeneration must be balanced inside the organelle. It has been proposed that compartmentalized glycolysis emerged to facilitate adaptation to different environmental conditions [9]. Other metabolic pathways that are compartmentalized inside glycosomes include the pentose phosphate pathway (PPP), nucleotide sugar biosynthesis, nucleotide biosynthesis and salvage, lipid synthesis, and fatty acid β-oxidation, probably due to their connection to glycolysis [10].

Gluconeogenesis (GNG) is a metabolic pathway that results in the generation of glucose from non-carbohydrate carbon substrates such as glycerol, lactate or glucogenic amino acids. In principle, it is the reverse of glycolysis, as many glycolytic enzymes are reversible, the direction depending on substrate and product concentrations. Only two steps are thought to be unique to GNG in protozoa; requiring fructose-1,6-bisphosphatase (FbPase) and phosphoenolpyruvate carboxykinase activity [11]. PCF *T*. *brucei* display GNG capacity fed by proline [12] but GNG was thought to be absent from BSF *T*. *brucei*, since FbPase activity was not detected [13]. Metabolomic analysis with labelled glucose also failed to reveal evidence for GNG [11]. Consequently, it is often stated that BSF trypanosomes depend ‘exclusively’ [14,15,16,17] or ‘entirely’ [18,19] on glycolysis, using glucose as a substrate, for ATP production (reviewed in [20,21]). For this reason, the glycolytic pathway has been considered to be a promising target for antitrypanosomal drug discovery [6]. On the other hand, Ryley [22] reported utilization of glycerol for respiration by both BSF and PCF *T*. *b*. *rhodesiense* in the early 1960s. Furthermore, glycerol has been used routinely to sustain trypanosomes in glucose-free media for several hours in radiolabelling experiments [23]. We also recently reported the use of glycerol for ATP production by BSF *T*. *brucei* [24].

There are thought to be five copies of each trypanosome hexose transporters gene, THT1 (Tb927.10.8440–8480) and THT2 (Tb927.10.8490–8530), arranged in an array in the *T*. *b*. *brucei* strain 927 reference genome, however the number of copies is variable across different strains [25]. The two gene types are similar, containing some identical domains. THT1 transcripts are substantially more abundant in BSF cells relative to PCF cells, while in PCF cells, THT2 transcripts are substantially more abundant than THT1 transcripts [26,27]. Specific regions in the 3’-untranslated regions contribute to this stage-specific expression pattern [28], while only THT2 transcripts are upregulated after glucose depletion [27]. The THTs comprise twelve trans-membrane domains and a cysteine-rich loop [26]. They are closely related to mammalian hexose transporters, although some substrate selectivity was observed [29]. Overall, the substrate selectivity of THT1 and THT2 is similar, but THT1 is a high capacity, low affinity transporter, whereas THT2 is a lower capacity, higher affinity transporter; this may reflect the conditions under which each of the proteins is expressed (reviewed in [26]).

Here, we explore glycerol utilization for GNG in *T*. *brucei* depleted of THTs, and find GNG operating even in wild-type cells that have access to glycerol. Carbons from stable isotope labelled glycerol are detected in sugar phosphates, PPP intermediates, VSG glycans and other metabolites. We also detect robust FbPase activity, even after deletion of the annotated FbPase gene. Thus, contrary to prior thinking, GNG is available to mammalian stage *T*. *brucei* and may operate in tissue environments where glycerol is available. This metabolic flexibility may be essential for adaptation to environmental conditions and survival in mammalian host tissues.

## Results

### THT1 and THT2 are plasma membrane-localised and life cycle stage-regulated

**Fig 1:**
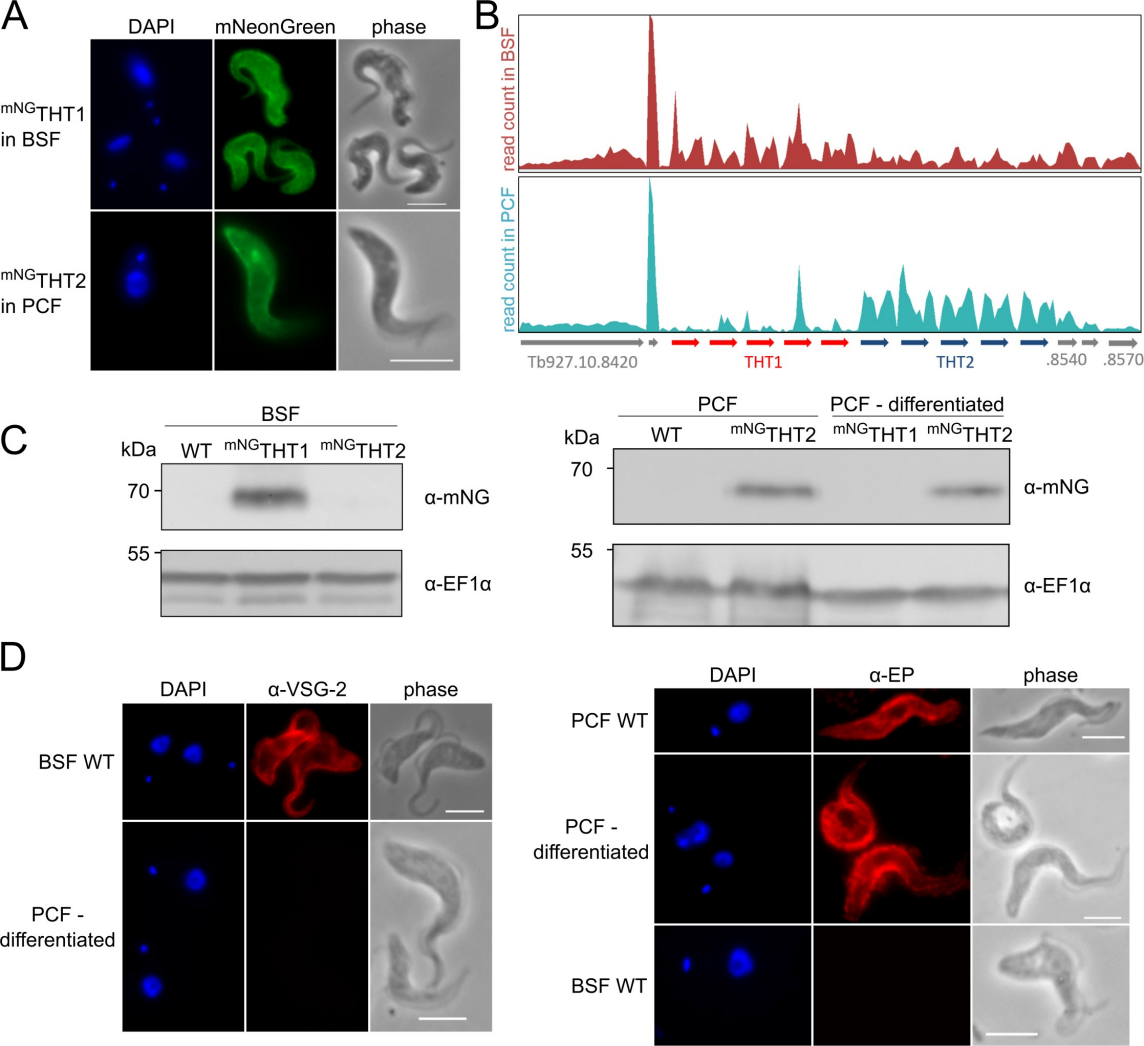
Glucose transporters localise to the cell surface and are developmentally regulated. (A) Fluorescence microscopy analysis. ^mNG^THT1 and ^mNG^THT2 localise to the cell surface in bloodstream and insect stage *T*. *brucei*, respectively, as shown by direct mNeonGreen (mNG) fluorescence. (B) Differential RNA-seq expression analysis of *THT1* and *THT2* genes in BSF and PCF showing developmental control. Total number of reads at the *THT1/THT2* locus, applying an uniqueness filter of MapQ>1, are depicted using the Artemis genome browser [66]; raw transcriptomic data was derived from Hutchinson *et al* [30]. (C) Western blot analyses indicating stage specific expression of *in situ* tagged ^mNG^THT1 and ^mNG^THT2 in BSF (left) and PCF and cells differentiated in DTM medium (right). WT, wild-type; EF1α serves as a loading control. (D) Differentiation in DTM medium of ^mNG^THT expressing cells (^mNG^THT2 cells shown) was validated by immunofluorescence microscopy with stage specific surface markers, VSG-2 for BSF and EP procyclin for PCF. In A and D, DNA was counter stained with DAPI; Scale bars 5 μm.

*T*. *brucei* hexose transporter 1 (THT1) transcripts are substantially more abundant than THT2 transcripts in the bloodstream stage, whereas the situation is reversed in the insect-stage [27]. Both proteins are thought to be localized to the plasma membrane. To determine whether this is indeed the case, we assembled strains that express an *N*-terminal mNeonGreen (mNG) tagged copy of each gene type; tags were inserted at the native loci. Both proteins were detected by direct fluorescence microscopy. ^mNG^THT1 was localised on the surface plasma membrane of bloodstream form cells, while ^mNG^THT2 was on the surface of insect stage cells (Fig 1A). To assess transcript levels, we analyzed our recently published transcriptome data from bloodstream and insect life cycle stages [30]. The region encompassing the five copies of THT1 and the five immediately adjacent copies of THT2 is shown in Fig 1B and confirms higher THT1 transcript expression in the bloodstream stage (Fig 1B, top panel) and almost exclusive THT2 expression in the insect stage (Fig 1B, lower panel). As noted above, specific regions in the 3’-untranslated regions contribute to this stage-specific expression pattern [28].

To explore developmental controls at the protein level, we assembled an additional BSF strain that expressed ^mNG^THT2. Bloodstream strains expressing either ^mNG^THT1 or ^mNG^THT2 were differentiated to insect stage cells. Subsequent protein blotting revealed an expression pattern that mirrored the transcript levels seen above. Specifically, ^mNG^THT1 was expressed primarily in bloodstream form cells, while ^mNG^THT2 was expressed primarily in insect stage cells (Fig 1C and 1D). Thus, we demonstrate plasma membrane localization of both hexose transporters and confirm the expected life-cycle stage regulated controls at both the transcript and protein levels.

### The hexose transporters are required for glucose uptake and viability

**Fig 2:**
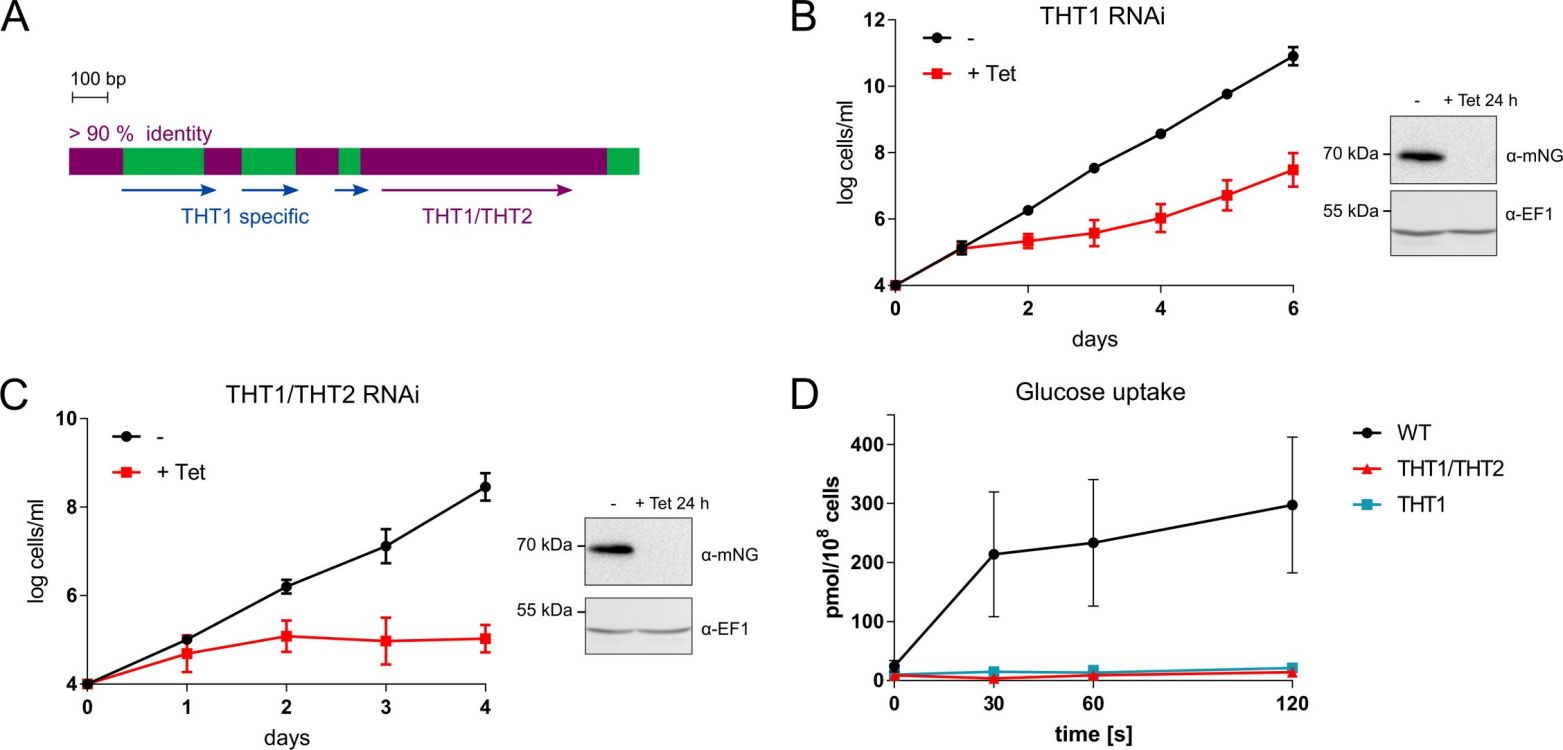
The glucose transporters are essential for growth *in vitro*. (A) The scheme shows the relationship between *THT1* and *THT2* genes. >90% identical regions are depicted in plum and *THT*-specific regions are in green. The arrows indicate the regions used for RNAi. (B) The growth curves show the THT1 RNAi cell line without and with tetracycline induction. Data points are means of two independent experiments with two biological replicates, error bars, SD. The protein blot shows depletion of native tagged ^mNG^THT1 in the same cell line 24 h after tetracycline induction. (C) The growth curves show THT1/THT2 RNAi cell line without and with tetracycline induction. Data points are means of two independent experiments with two biological replicates, error bars, SD. The protein blot shows depletion of native tagged ^mNG^THT1 in the same cell line 24 h after tetracycline induction. (D) An uptake assay for [^14^C]2-deoxyglucose showing that this glucose analogue is taken up by wild-type (WT), but not THT1 and THT1/THT2 induced RNAi cells after 3 days tetracycline induction (n = 3). Error bars, SD.

THT1 and THT2 are closely related but contain multiple diverged domains (Fig 2A). For functional assessment of THT1, we assembled an RNA interference (RNAi) construct targeting the unique portions of the THT1 genes (Fig 2A) and used this construct to assemble knockdown strains that also expressed a native tagged copy of ^mNG^THT1. We then induced specific knockdown of THT1, which revealed a substantial growth defect; efficient ^mNG^THT1 knockdown was confirmed by protein blotting (Fig 2B). Although growth was perturbed by THT1-specific knockdown, cell growth continued, and increased over time (Fig 2B), possibly due to loss of the RNAi construct [31] or low-level THT2 expression (see Fig 1B and S1A Fig). We, therefore, assembled a second RNAi construct targeting the shared portions of both THT1 and THT2 simultaneously, and used this construct to assemble knockdown strains that again expressed a native tagged copy of ^mNG^THT1. We induced simultaneous knockdown of THT1 and THT2, which in this case revealed a severe and more pronounced growth defect; efficient ^mNG^THT1 knockdown was once again confirmed by protein blotting (Fig 2C). We also demonstrated the capacity for ^mNG^THT2 knockdown using this approach in insect stage *T*. *brucei* (S1B Fig). Failure to grow after simultaneous THT1 and THT2 knockdown (Fig 2C) indicated that the hexose transporters are essential for continued growth in BSF trypanosomes. This is consistent with the idea that these transporters are essential uptake routes for glucose and also that glycolysis is required for ATP production in these cells [4].

**Fig 3:**
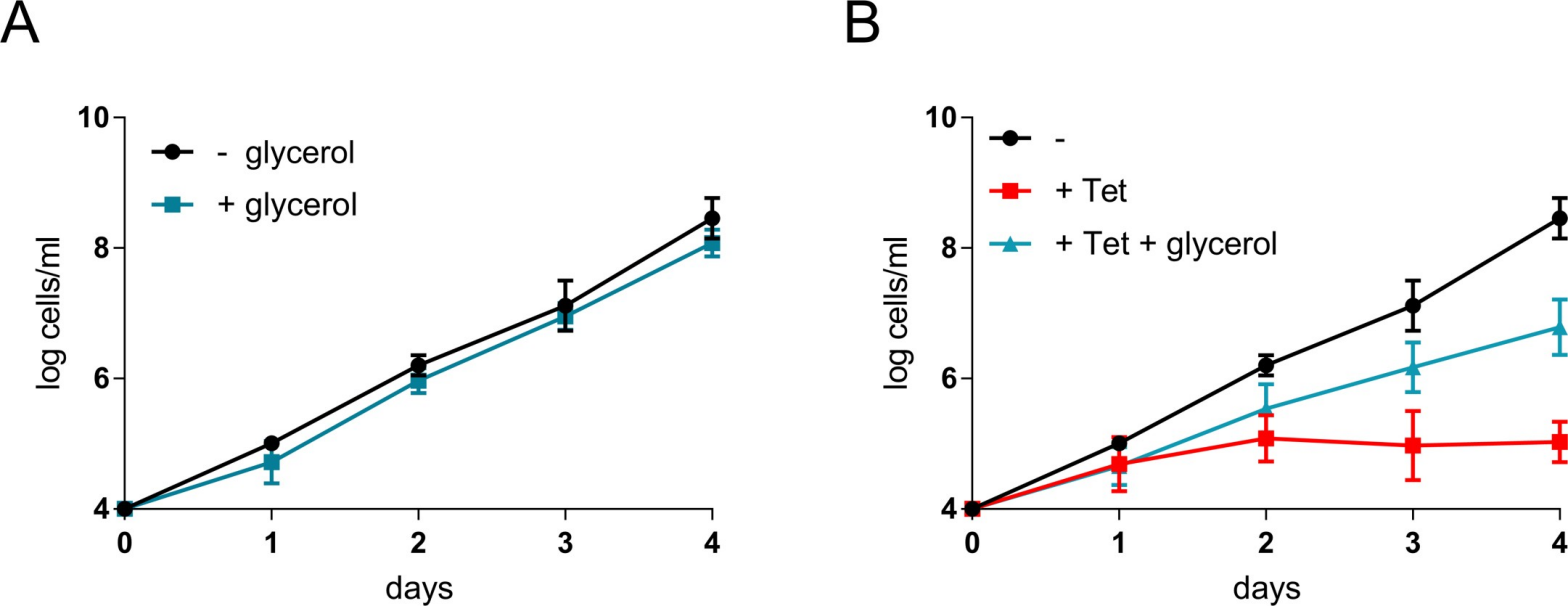
Glycerol rescues bloodstream form trypanosomes lacking glucose transporters. (A) Growth curve showing impact of glycerol without hexose transporter knockdown. Addition of 5 mM glycerol triggers non-significant, but consistent mild growth defect. Data points are means of two independent experiments with two biological replicates. Error bars, SD. (B) Growth curve showing impact of glycerol on THT1/THT2 knockdown cells. 5 mM glycerol partially rescues the knockdown-associated growth defect. Data points are means of two independent experiments with two biological replicates. Error bars, SD.

To directly measure glucose uptake in cells depleted for THT1 or for both THT1 and THT2, we assessed the accumulation of the radiolabelled glucose analogue, 2-^14^C(U)-deoxyglucose, in wild-type and knockdown strains; knockdown was carried out in the presence of 5 mM glycerol (see Fig 3 below). We observed no uptake of label in cells incubated at 4°C but robust accumulation of label in wild-type cells incubated at 37°C over a 2 min time-course; 428 ± 211 pmol/min/10^8^ cells (Fig 2D). In contrast, accumulation of label was 20-fold lower in knockdown cells; THT1-knockdown yielded only marginally (1.5-fold) higher labelling relative to THT1/THT2 knockdowns (Fig 2D).

### Glycerol supplementation rescues the THT-knockdown defect

The growth medium typically used to propagate BSF *T*. *brucei* in culture is rich in (25 mM) glucose [32]. This HMI-11 growth medium may fail to reflect the full range of metabolic pathways available to these parasites in their natural host environment. We were particularly interested in the possible utilization of glycerol as a carbon source, since *T*. *brucei* express aquaglyceroporins (AQPs) [24,33]. These AQPs appear to be important to remove glycerol from the cells, a product of glycolysis under anaerobic conditions [34]. On the other hand, we recently demonstrated that *T*. *brucei* can use glycerol as an energy source, in an AQP-dependent manner [24]. In addition, *T*. *brucei* have recently been shown to occupy adipose tissue in a mouse model [1], a tissue where the glycerol concentration is four-fold higher than in blood (~200 μM) [35,36].

We, therefore, exploited the THT-knockdown strains to explore the ability of *T*. *brucei* to use and to grow on glycerol as a carbon source. Supplementation of the standard HMI-11 growth medium with 5 mM glycerol had little impact on *T*. *brucei* growth (Fig 3A). Notably though, the same supplementation, of cells in which THT1 and THT2 had been simultaneously knocked down, substantially rescued the severe growth defect observed above (Fig 3B). These results indicate that glycerol can serve as an alternative carbon source for BSF *T*. *brucei*. It was previously observed that replacement of glucose with glycerol triggered differentiation of BSF into PCF [37], however we did not observe such a phenotype with THT knockdown cells; all cells continued to express variant surface glycoprotein 2 (VSG-2) and no cells expressed EP procyclin following 5 days of knockdown (S1C Fig).

### Exogenous glycerol is a substrate for gluconeogenesis

We next employed a liquid chromatography–mass spectrometry (LC-MS) metabolomics approach to explore the utilization of stable heavy isotope labelled glycerol as a carbon source in bloodstream form trypanosomes. For these experiments, wild-type and THT1/THT2 knockdown cells were grown in HMI-11 medium supplemented with 5 mM U-^13^C_3_-glycerol (glycerol with heavy isotope labelled carbon in all three positions) for three days; knockdown was induced over the three-day time-course in the latter case. Both samples cultured without any ^13^C label, but with 5 mM supplementary glycerol in the case of the knockdowns, were grown and analysed in parallel. Subsequent metabolomic analyses allowed us to detect a number of intermediates of metabolism and also to trace the ^13^C-labelled metabolites.

**Fig 4:**
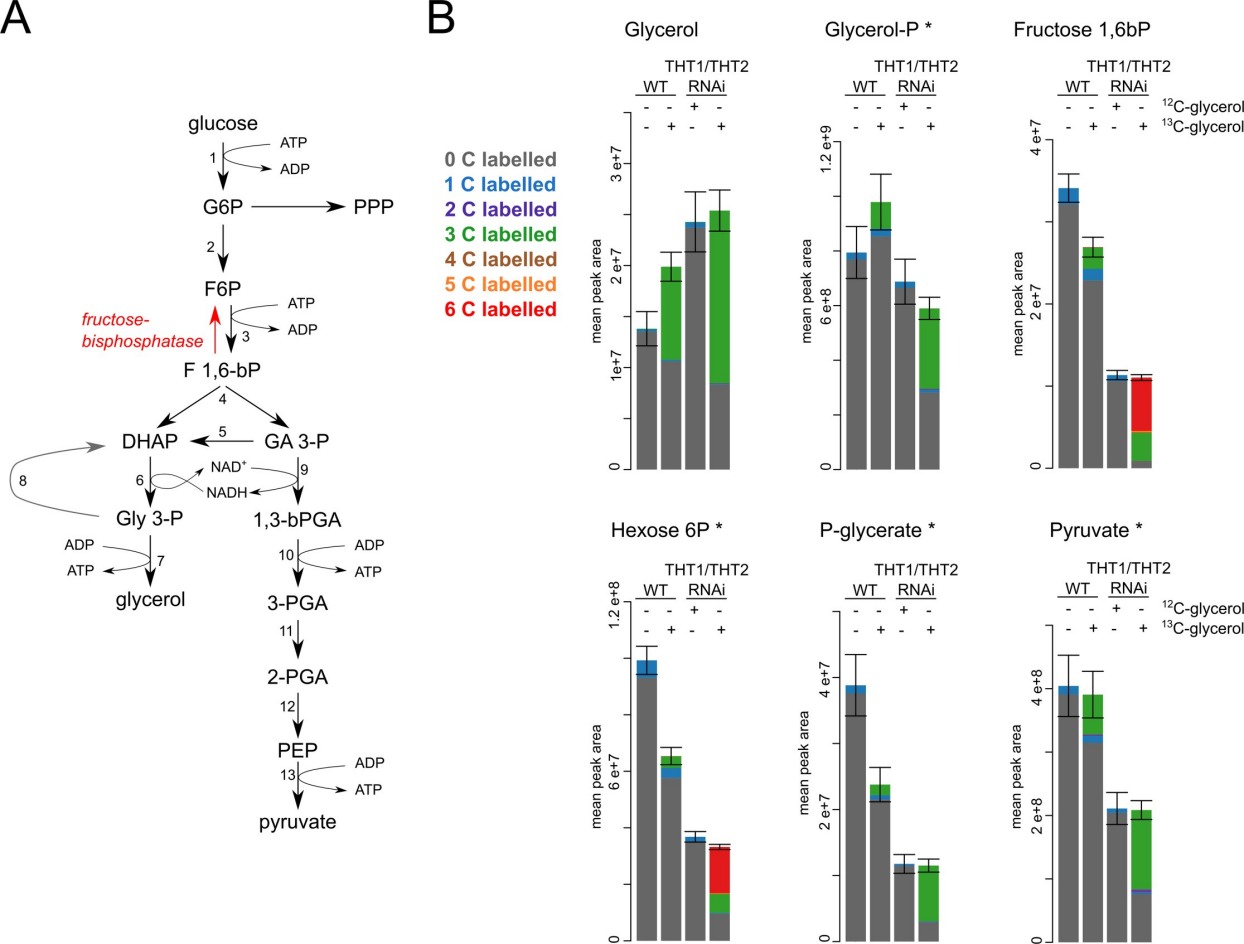
Gluconeogenesis in trypanosomes growing on glycerol. (A) A scheme of glycolysis and gluconeogenesis with highlighted fructose-1,6-bisphosphatase. PPP, pentose phosphate pathway; 1, hexokinase; 2 glucose-6-phosphate isomerase; 3, phosphofructokinase; 4, fructose-bisphosphate aldolase; 5, triose-phosphate isomerase; 6, glycerol-3-phosphate dehydrogenase; 7, glycerol kinase; 8, mitochondrial glycerol-3-phosphate dehydrogenase; 9, glyceraldehyde-3-phosphate dehydrogenase; 10, phosphoglycerate kinase; 11, phosphoglycerate mutase; 12, enolase; 13, pyruvate kinase. (B) Metabolites from glycolysis/gluconeogenesis as detected in the LC-MS analysis. The size of the bars represents the total abundance, and coloured parts indicate ^13^C labelling as depicted in the key. The samples are from WT cells, WT grown in ^13^C-glycerol, THT1/THT2 RNAi grown in ^12^C-glycerol and THT1/THT2 RNAi grown in ^13^C-glycerol. Glucose was also present for all experiments. Natural abundance of ^13^C is 1%, hence the 1C labelling in ‘un-labelled’ samples that is proportional to the number of carbons in each metabolite. * identity of these metabolites was confirmed using a match with a standard.

Fig 4A indicates key steps in glycolysis and in the reverse pathway of gluconeogenesis, including the metabolites that we detected and analysed by LC-MS. In terms of the exogenous ^13^C_3_ labelled glycerol, we see that this is taken up by both wild-type and THT1/THT2 knockdown cells (Fig 4B; glycerol panel, green); two thirds (67%) of the glycerol in the knockdown cells was ^13^C_3_ labelled. Notably, the glycerol content was increased in all three cases where exogenous glycerol was supplied, particularly following knockdown (Fig 4B). Next, in the knockdown cells, we can see that the labelled glycerol was phosphorylated by glycerol kinase to form glycerol 3-phosphate (Fig 4B; glycerol-P panel, green); half (50%) of the glycerol-phosphate in the knockdown cells was ^13^C_3_ labelled. Labelled glycerol-phosphate was then channelled to further gluconeogenic intermediates. For fructose 1,6-bisphosphate (Fig 4B; fructose 1,6bP panel), almost a third (31%) was ^13^C_3_ labelled (green) and more than half (59%) was fully ^13^C_6_ labelled (red). For hexose 6-phosphates (Fig 4B; hexose 6P panel), one fifth (20%) was ^13^C_3_ labelled (green) and almost half (49%) was fully ^13^C_6_ labelled (red). For 2/3-phosphoglycerate (Fig 4B; P-glycerate panel, green), almost three quarters (74%) was ^13^C_3_ labelled, and similar patterns were observed in pyruvate (Fig 4B; pyruvate panel, green) and phosphoenolpyruvate (S2 Fig). These labelled intermediates shown in Fig 4B were also detected, albeit at reduced levels (^13^C_3_-labelled part comprised 10, 6, 7 and 16%, respectively), in wild-type cells. Thus, even wild-type *T*. *brucei* take up glycerol, when available, and use it as a substrate for gluconeogenesis.

*T*. *brucei* appear to prefer to use glucose, when available, as a carbon source. The cells analysed here that use mostly glycerol instead of glucose do grow at a slower rate (Fig 3B). When glycerol is used, pathway intermediates drop to approximately one third of the levels observed in wild-type cells grown on glucose; fructose 1,6-bisphosphate drops to 32% (Fig 4B; fructose 1,6bP panel), hexose 6-phosphates drop to 33% (Fig 4B; hexose 6P panel) and 2/3-phosphoglycerate drops to 29% (Fig 4B; P-glycerate panel). Even wild-type cells display decreased levels of glycolytic intermediates when grown in the presence of glycerol, compared to levels observed in wild-type cells grown on glucose: fructose 1,6-bisphosphate, 79% (Fig 4B; fructose 1,6bP panel); hexose 6-phosphates, 66% (Fig 4B; hexose 6P panel); phosphoglycerate, 61% (Fig 4B; P-glycerate panel). Thus, glycerol both inhibits the utilization of glucose for glycolysis and is effectively channelled into gluconeogenesis under glucose deprivation.

### *De novo* synthesised glucose 6-phosphate is utilised in other metabolic pathways

**Fig 5:**
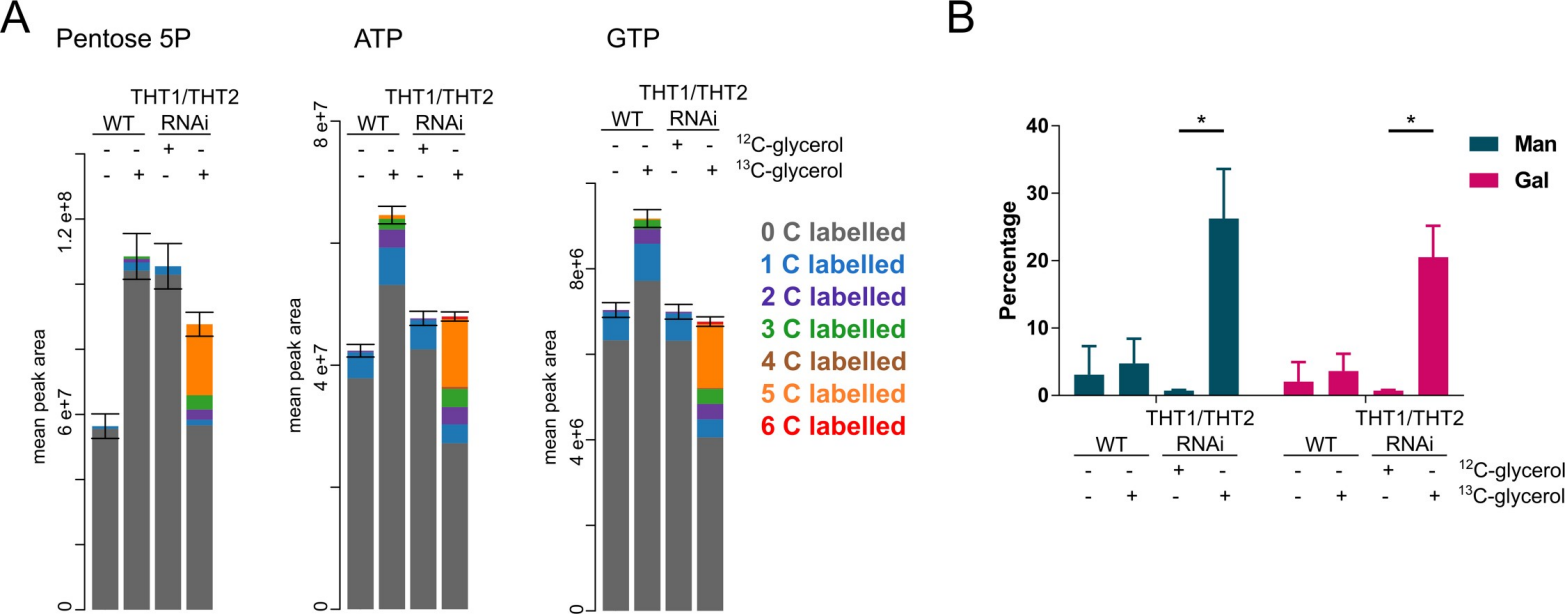
Carbons from glycerol are incorporated into PPP intermediates and VSG glycans. (A) ^13^C from glycerol is incorporated into PPP intermediates, such as pentose 5-phosphate which is further used for ATP and GTP synthesis. The samples and visualization are as in Fig 4B. (B) VSG was purified from wild-type and THT1/THT2 RNAi knockdown parasites grown with and without ^13^C glycerol, as indicated, and subjected to monosaccharide compositional analysis by GC-MS. Following correction for natural isotope abundance, the proportion of VSG-derived mannose and galactose residues containing ^13^C carbon from glycerol were determined. For all analyses, n = 3; * *p* < 0.0001, Student’s *t*-test.

We find that glucose 6-phosphate (G6P) synthesised from ^13^C_3_-glycerol is utilised in additional metabolic pathways, such as the pentose phosphate pathway (PPP) and for protein glycosylation. The oxidative branch of the PPP is indispensable in BSF *T*. *brucei*, being the main production route of reduced NADPH, a component essential for oxidative stress defence [38]. G6P is used as a substrate and since the non-oxidative branch of the pathway is missing in BSF *T*. *brucei*, the final end product is ribose 5-phosphate [39]. 25% of pentose phosphate is fully ^13^C_5_ labelled in trypanosomes grown on glycerol (Fig 5A), and ribose 5-phosphate is further incorporated into ATP and GTP, as demonstrated with 23% and 22%, respectively, being ^13^C_5_ labelled (Fig 5A). We did not detect sedoheptulose 7-phosphate, octulose 8-phosphate or erythrose 4-phosphate in the LC-MS data suggesting that transketolase activity is absent, as reported previously for BSF *T*. *brucei* [13,40]. Hence, the non-oxidative branch of the PPP is not activated under hexose-transporter knockdown and glycerol-replete conditions and the labelling pattern of the glycolytic end products is consistent with this activity being absent, i.e. all three or no carbons are labelled in phosphoglycerate, phosphoenolpyruvate and pyruvate; carbon shuffling would have been indicative of the non-oxidative branch of the PPP (Fig 4, S2 Fig, S1 Table).

^13^C carbons from glycerol are incorporated into numerous additional metabolic intermediates under hexose-transporter knockdown and glycerol-replete conditions (S1 Table). Alanine is produced as an end product from pyruvate, and ^13^C_3_-alanine comprises 63% of the total following THT1/THT2 knockdown; 17% in wild-type cells (S2 Fig, S1 Table). Orotate, an intermediate of pyrimidine synthesis, comprises 50% fully labelled following THT1/THT2 knockdown (S2 Fig), and the label is further incorporated into pyrimidines. For example, UDP contains a substantial proportion of ^13^C_2_ from orotate (32%) and ^13^C_5_ from ribose 5-phosphate (14%), resulting in ^13^C_7_ (10%, S2 Fig). Glycerol is also incorporated into glycerophospholipids, e. g. glycerol-3-phosphoinositol, glycerol-3-phosphocholine and glycerol-3-phosphoethanolamine (S2 Fig). We also detected a substantial proportion of *N*-acetylglucosamine with two, three, six and eight carbons labelled following THT1/THT2 knockdown (S2 Fig).

G6P is the sole sugar precursor of UDP-galactose (UDP-Gal) [41] and, in the absence of exogenously added mannose, of GDP-mannose (GDP-Man) [42,43]. These nucleotide sugars are the Gal and Man donors for the glycosyltransferases involved in protein *N*-glycosylation and glycosylphosphatidylinositol (GPI) anchor addition and processing in the parasite, including for the major cell surface component, the variant surface glycoprotein (VSG). Thus VSG is a convenient terminal metabolite with which to assess GNG. Soluble form VSG was purified from wild-type and THT1/THT2 knockdown parasites grown in the presence of ^13^C glycerol and subjected to monosaccharide composition analysis by GC-MS, following methanolysis and trimethylsilyl- (TMS-) derivatization. Electron impact mass spectra of methyl-glycoside TMS derivatives produce an intense [(CH_3_)_3_Si-**O-CH = CH-O**-Si(CH_3_)]^+^ fragment ion at *m/z* 204, where the sugar-derived atoms are indicated in bold. The natural relative (to *m/z* 204) abundance of *m/z* 206 from this composition is 8.6%. We therefore interpret relative abundance values of *m/z* 206 of >8.6% as being synonymous with the appearance of ^13^C from glycerol into the respective monosaccharides (S3 Fig). Our sample analyses indicated that 26 ± 7% of Man and 21 ± 5% of Gal was ^13^C labelled following THT1/THT2 knockdown in the presence of ^13^C glycerol, whereas labelling in all the other samples was not statistically significant (Fig 5B). The labelling of Man and Gal in the VSG *N*-linked and GPI glycans from ^13^C glycerol shows that G6P from GNG is used to drive terminal metabolite biosynthesis, as well as intermediate metabolism.

### GNG-specific fructose-1,6-bisphosphatase activity from *T*. *brucei*

Most of the enzymatic steps of glycolysis are reversible and used in GNG. The fructose 6-phosphate to fructose 1,6-bisphosphate step, catalyzed by phosphofructokinase, is often irreversible, however, such that the reverse reaction requires a GNG-specific fructose-1,6-bisphosphatase (FbPase, EC 3.1.3.11, see Fig 4A) activity. Thus, utilization of glycerol to generate hexose 6-phosphates typically requires FbPase activity, and our metabolomics data above suggest the presence of such an activity in *T*. *brucei* grown on glycerol and even in wild-type cells. It was failure to detect this activity that previously suggested the absence of GNG in *T*. *brucei*, however [13]; with a limit of detection, 0.5 nmol/min/mg protein.

**Fig 6:**
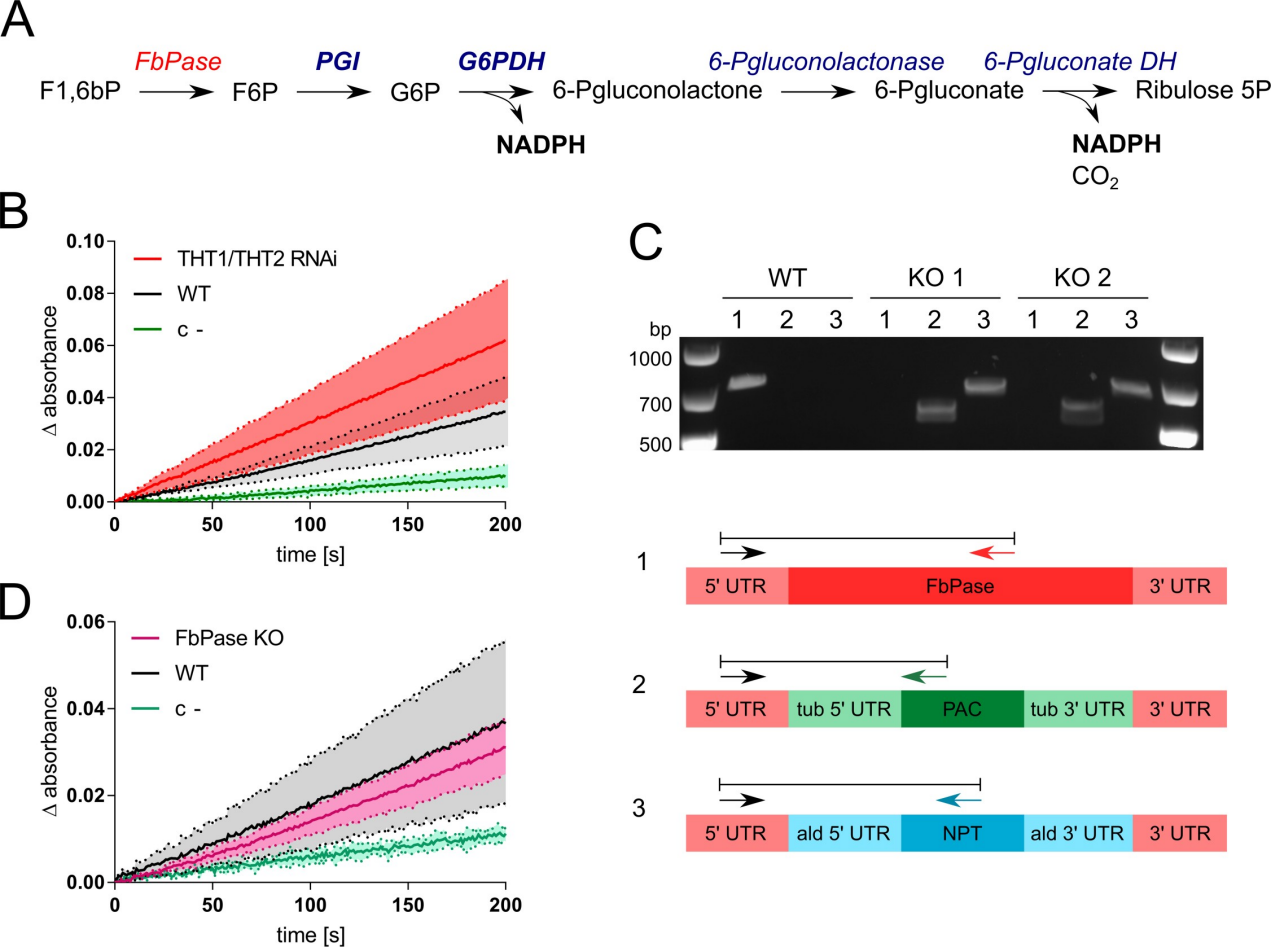
Fructose-1,6-bisphosphatase activity in bloodstream form trypanosomes. (A) A scheme showing the coupled enzymatic assay used for fructose-1,6-bisphosphatase (FbPase) activity detection. F1,6bP is added to cell extract, and if FbPase is present, it converts F1,6bP into F6P. F6P is converted into G6P by glucose-6-phosphate isomerase (PGI) and further into 6-phosphogluconolactone by glucose-6-phosphate dehydrogenase (G6PDH) producing NADPH. Further, 6-phosphogluconate is made by 6-phosphogluconolactonase, followed by a 6-phosphogluconate dehydrogenase reaction resulting in ribulose 5-phosphate and additional NADPH. PGI and G6PDH were added to the reaction, while the latter two enzymes are present in the cell extract. NADPH production is detected spectrophotometrically. (B) FbPase activity detected in WT (no glycerol in growth medium) and THT1/THT2 knockdown cells induced with tetracycline (plus 5 mM glycerol) for 3 days, and a negative control where F1,6bP was omitted. The lines show means of replicates (n = 3) and shaded areas indicate SD. The slopes measured for WT and THT1/THT2 knockdown cells are significantly different (*p* < 0.0001, linear regression). (C) Validation of the FbPase (Tb927.9.8720) knockout (KO); two independent populations. The gel shows the PCR assays and the schematic maps indicate the native FbPase gene (lane 1), and alleles after precise replacement with a *PAC* (lane 2) or *NPT* cassette (lane 3). (D) FbPase activity detected in FbPase KO cells. The lines show means of replicates (n = 2) for two independent FbPase KO populations. Other details as in A-B above.

We measured FbPase activity using a coupled enzymatic assay (Fig 6A). FbPase enzyme activity was indeed detected in both wild-type cells grown in the absence of glycerol and hexose transporter knockdown cells grown in the presence of glycerol, at 10.6 ± 4.5 nmol/min/mg protein (n = 3) and 15.6 ± 3.5 nmol/min/mg protein (n = 3), respectively (Fig 6B). These measurements correspond with the metabolomic data, since FbPase activity is required for the incorporation of carbons from glycerol into G6P. Thus, BSF *T*. *brucei* has the enzymatic capacity to perform GNG, and the activity of the pathway appears to be specifically increased under hexose-transporter knockdown and glycerol-replete conditions.

*T*. *brucei* express a glycosomal FbPase orthologue (Tb927.9.8720) [7] in both the BSF and PCF life-cycle stages [44]. To further explore the source of the FbPase activity detected above, we generated Tb927.9.8720-null cells in which the protein coding sequences were precisely removed (Fig 6C), using a Cas9-based editing approach [45]. An FbPase activity at 11.2 ± 7.1 nmol/min/mg protein (n = 2 replicates of 2 populations) was also detected in these null cells grown in the absence of glycerol (Fig 6D). Thus, *T*. *brucei* bloodstream form cells express a Tb927.9.8720-independent source of FbPase activity.

### Metabolic compensations for gluconeogenesis

**Fig 7:**
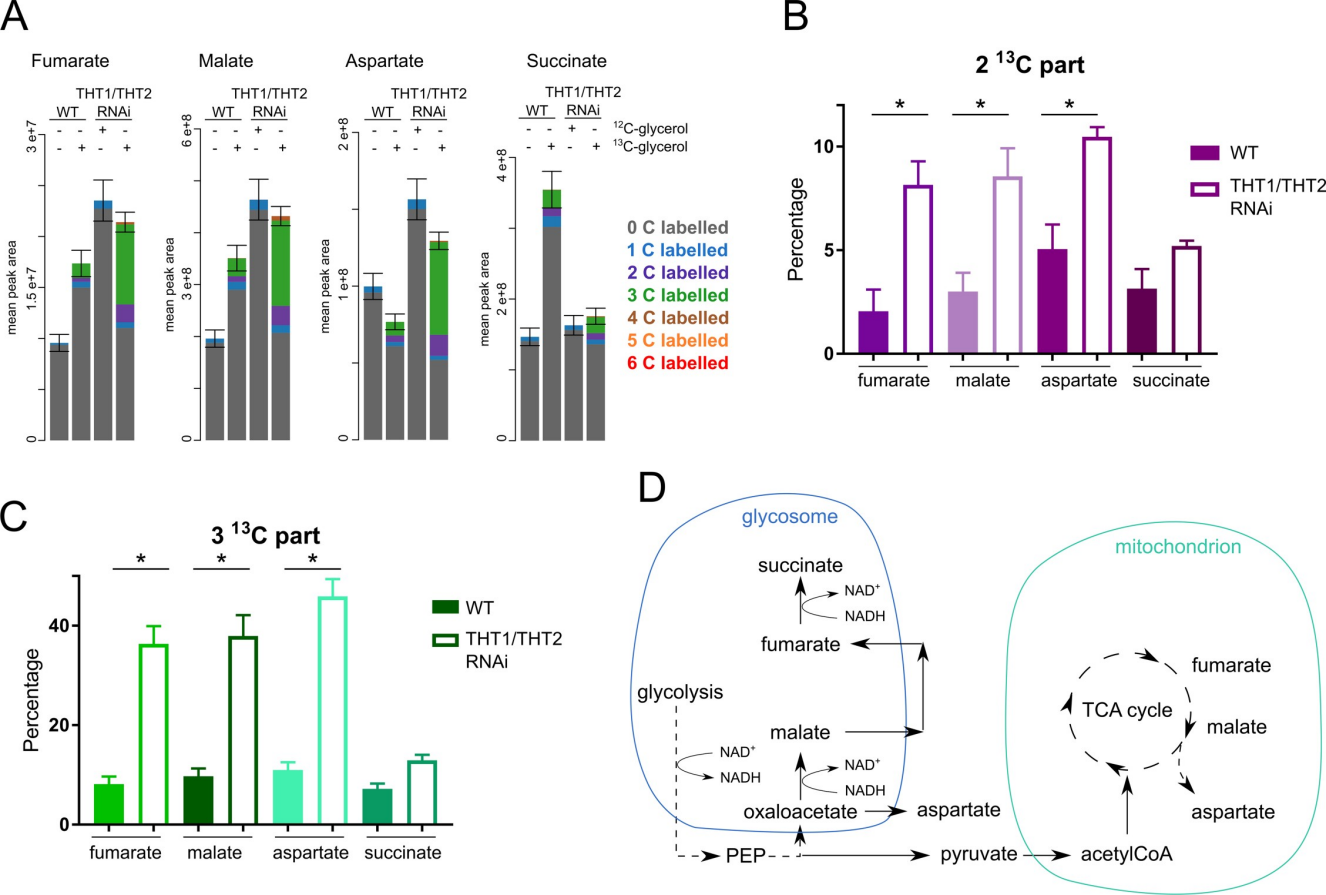
Metabolic adjustments of trypanosomes growing on glycerol. (A) Profiles of fumarate, malate, aspartate and succinate as detected by LC-MS metabolomics. Samples and visualization as in Fig 4B. (B) Proportion of these metabolites with two ^13^C labelled carbons in WT and THT1/THT2 RNAi cells, * *p* < 0.001. (C) Proportion of these metabolites with three ^13^C labelled carbons in WT and THT1/THT2 RNAi cells, * *p* < 0.001. (D) Scheme indicating potential explanation for the labelling patterns observed. The ^13^C_2_ part may be produced by the TCA cycle activity fed by ^13^C_2_ labelled acetyl-CoA, advantageous for additional ATP production. The ^13^C_3_ part could be produced in the glycosomal succinate shunt, which is activated in order to maintain the NAD^+^/NADH balance inside the organelle.

The metabolomic analysis indicated other notable labelled metabolites under hexose-transporter knockdown and glycerol-replete conditions, possibly indicating metabolic compensations for GNG (Fig 7A). Specifically, the total amount of fumarate and malate is two-fold increased under these conditions (Fig 7A), suggesting higher flux in both the TCA cycle and the succinate shunt. Between 2–5% of malate, fumarate, and aspartate are ^13^C_2_ labelled in wild-type cells, which increases three fold (to 8–10%) under hexose-transporter knockdown and glycerol-replete conditions (Fig 7A and 7B). This may be due to increased activity of the tricarboxylic acid (TCA) cycle, since ^13^C carbons from glycerol are passed into ^13^C_2_ labelled acetyl-CoA, which is then incorporated into TCA cycle intermediates (Fig 7D). Acetyl-CoA was not detected in the LC-MS analysis, but *N*-acetyl-lysine, GlucNAc (S2 Fig, S1 Table) and other intermediates containing ^13^C_2_ indicate labelling from acetyl-CoA. Operation of the TCA cycle was also suggested recently for adipose tissue resident *T*. *brucei* [1].

Under glycolysis, dihydroxyacetonephosphate (DHAP) is converted into glycerol 3-phosphate (Gly 3-P) by glycosomal glycerol-3-phosphate dehydrogenase (G3PDH), producing oxidised NAD^+^, but during GNG this source of NAD^+^ is lost and must be compensated for. Since the glycosomal membrane is impermeable to large metabolites such as NAD(H), NAD/H, recycling has to be balanced within the glycosome [46]. Upregulation of the glycosomal succinate shunt could compensate for this deficiency through malate dehydrogenase activity and NADH dependent fumarate reductase activity. Consistent with this view, we observe similar labelling for malate, fumarate and aspartate (Fig 7A), with relatively small proportions (8–11%) of these metabolites containing ^13^C_3_ label (Fig 7C) in wild-type *T*. *brucei* in the presence of glycerol, which increases under hexose-transporter knockdown and glycerol-replete conditions; approximately three-fold, and reaching 36–47% (Fig 7C). These metabolites are likely products of phosphoenolpyruvate (PEP) being converted into oxaloacetate, and further into malate, fumarate and succinate. This suggests upregulation of the glycosomal succinate shunt (Fig 7D) to compensate for NAD/H balance in the glycosome when glycosomal G3PDH is inactive (oxidising NADH to NAD^+^); although relatively low succinate labelling suggests that succinate can also be derived from another pathway.

Thus, since use of glycerol produces only a single molecule of ATP per molecule of glycerol when GNG is operative, the cells may compensate by producing ATP in the mitochondrion, either by the TCA cycle activity or by acetate:succinyl-CoA shunt via succinyl-CoA synthetase converting succinyl-CoA into succinate [47].

## Discussion

It has been thought for decades that mammalian forms of *T*. *brucei*, the causative agents of African sleeping sickness in humans and nagana in cattle, are exclusively dependent on glycolysis, using glucose as a substrate, for ATP production. We now provide evidence that these cells can utilise glycerol for gluconeogenesis (GNG) and for ATP production. Thus, metabolism in these parasites is not as simplified and reduced as had been thought. Indeed, we demonstrate some GNG even in wild-type cells grown on glycerol, even when glucose is also available. Metabolism may indeed be simple and dependent on glycolysis in blood, but it now seems likely that GNG can be activated in different environments.

Creek and colleagues [11] utilised metabolomic analyses to demonstrate extensive utilization of glucose in a wide range of metabolic pathways. We now report the utilization of glycerol for many of those same pathways. FbPase is considered to be one of the rate-limiting activities of GNG in other systems [48], but this activity has not previously been detected in *T*. *brucei* and therefore kinetic parameters have not been established. An annotated FbPase (Tb927.9.8720) is present in trypanosome glycosomes [7] and is expressed at similar protein levels in both bloodstream and insect life-cycle stages [44], the transcript levels are similarly not significantly different between culture, blood, or adipose tissue derived BSF [1]. We now show that FbPase activity is present in BSF *T*. *brucei* cells and increases when GNG is activated. We also show, however, that this activity is Tb927.9.8720-independent. Thus, the dephosphorylation of F1,6bP to F6P in *T*. *brucei* may require reversal of the phosphofructokinase reaction or sedoheptulose-1,7-bisphosphatase (Tb927.2.5800) activity [49].

In addition to FbPase, phosphoenolpyruvate carboxykinase (PEPCK) is the other enzyme specific for GNG, and responsible for its regulation in mammals [48]; this enzyme is also known to be present and active in *T*. *brucei* [11]. As expected, a third GNG-specific enzyme in animals, glucose-6-phosphatase, is not present in *T*. *brucei*, nor in other protozoa, since loss of the phosphate group would allow free glucose to diffuse out of the cells [48]. Previous modelling and metabolomics of glycolysis suggested high reverse flux of aldolase [11], however, similar information is lacking for the other enzymes. *T*. *brucei* glycerol kinase is unique in its bidirectional activity, and is known to lack the classical allosteric regulation [50]. According to canonical biochemistry, glycolysis and GNG cannot operate simultaneously, and the exclusive regulatory mechanisms are well known in mammalian systems. However, this classical regulation is missing in the *T*. *brucei* enzymes [34,51]. Compartmentalization in glycosomes would not present a solution if both glycolysis and FbPase activity are localised inside glycosomes [7] and we cannot exclude futile cycling in *T*. *brucei*, which has been suggested in *Toxoplasma gondii* [52]. However, since we now demonstrate Tb927.9.8720-independent FbPase activity, further work will be required to determine whether this FbPase activity is indeed compartmentalized within glycosomes in *T*. *brucei*.

GNG is essential in other unicellular pathogens. It is vital for the mammalian stage of *Leishmania* due to the need for mannogen biosynthesis [53]. GNG is also vital for *Toxoplasma gondii*, regardless of whether infected host cells are rich or poor in glucose [52]. *Mycobacterium tuberculosis* is also dependent on GNG and harbours two independent FbPase genes [54]. GNG may be particularly important in mammalian form *T*. *brucei* to feed the essential pentose-phosphate and glycoprotein glycosylation pathways, since even if the cells produce ATP from the second half of glycolysis when glucose is limiting, G6P is required as a substrate for these other essential pathways.

Aquaglyceroporins (AQPs) transport glycerol, but have generally been considered important for glycerol efflux rather than acquisition [33]. AQPs can be used for glycerol uptake to fuel *T*. *brucei* metabolism, however [24]. Indeed, our current data are consistent with a report from 1962 on trypanosomes using glycerol as a substrate for respiration [22], and a report from 1977 on pyruvate production from glycerol [55]. Is glycerol the only GNG substrate in *T*. *brucei*? *Leishmania* can also use aspartate, alanine or lactate to feed GNG [56]. Although the same enzymatic repertoire is theoretically available in *T*. *brucei* [4], our findings suggest that amino acid supply in growth medium fails to fuel GNG in cultured bloodstream form *T*. *brucei*. On the other hand, glycerol may activate GNG, thereby allowing other substrates to be used.

Futile cycling between glycolysis and GNG would be energetically disadvantageous, but it is consistent with our observation that GNG is associated with a fitness cost. Depletion of triosephosphate isomerase, leading to production of one ATP per molecule of glucose, caused a severe growth defect in BSF *T*. *brucei* [57]. Similarly, one molecule of ATP is produced per molecule of glycerol when GNG is operative, while two molecules of ATP are produced per molecule of glucose in glycolysis. However, this may be compensated for by upregulating glycerol uptake. Decrease in glycolytic intermediates indicates decrease of flux in glycolysis, which may be a consequence of futile cycling. In addition, glycerol must be phosphorylated in the initial step of GNG, potentially inhibiting glycolysis by depleting the ATP required to drive the hexokinase and the phosphofructokinase reactions.

In parallel with GNG, we do see evidence for metabolic compensation to balance ATP production. Additional ATP may be produced in the mitochondrion in the acetate:succinyl-CoA shunt, the TCA cycle directly by succinyl-CoA synthetase, or indirectly by feeding the electron transport chain with reduced cofactors from the TCA cycle. Specifically, labelling patterns observed in fumarate, malate, and aspartate support activity of the TCA cycle, as proposed previously in adipose tissue trypanosomes [1]; pyruvate is converted into acetyl-CoA, which then introduces two labelled carbons into the TCA cycle. An alternative explanation for this labelling pattern is that the enzymes, malate dehydrogenase and fumarase, are working in both directions and shuffling carbons, as a consequence of GNG disrupting the usual metabolic steady state. The glycerol-derived ^13^C_3_ component of fumarate, malate, and aspartate; comprising about 8% in wild-type, increased three-fold following hexose transporter knockdown. This may reflect glycosomal succinate shunt activity or the reverse action of TCA cycle enzymes. Notably, increased activity of the glycosomal succinate shunt would also serve to regenerate NAD^+^ and maintain NAD^+^/NADH balance inside the glycosome.

Various mammalian host tissues may provide glycerol in quantities sufficient for GNG in trypanosomes. Infection in the cerebrospinal fluid is well known. However, little is known about parasite metabolism in the central nervous system and glycerol concentration is lower than in blood [36]. Adipose tissue contains glucose at concentrations about seven fold lower than in plasma [58] and glycerol at concentrations about four fold higher than in plasma [35,36], although these levels may be variable. Adipose tissue form trypanosomes may possess metabolically active or upregulated pathways, which are silent in the BSF, i.e. the TCA cycle and fatty acid β-oxidation [1], and we show here that GNG is active in the presence of glycerol. *T*. *brucei* are also present in skin, but their metabolism has yet to be scrutinised in this tissue [2,3]. Trypanosomes may never encounter environments completely lacking glucose under physiological conditions in mammalian hosts. Notably, in this regard, GNG is operative when both glucose and glycerol are present. Incomplete labelling in our glycerol-fed metabolomics analyses suggests that, although glucose uptake was below the detection limit of our assay in the absence of hexose transporters, some glucose was likely still imported by endocytosis. An ability to use both substrates may be crucial for adaptation to particular tissue environments or during transitions between tissues.

Our metabolomics analysis indicates that, in the presence of a suitable substrate, GNG does operate in mammalian form *T*. *brucei*. The pathway even operates, albeit at a relatively low level, in wild-type cells in the presence of glycerol. Cells that have limited access to glucose, in this case following hexose transporter knockdown, display a major increase in flux through GNG. We conclude that GNG in *T*. *brucei*, using glycerol taken up via aquaglyceroporins [24], could be important for colonization of, and survival in, different host tissue environments.

## Materials and methods

### Cell culture

*T*. *b*. *brucei* Lister 427 bloodstream form cells were cultured in the standard HMI-11 medium (Gibco), supplemented with 10% fetal bovine serum (Sigma-Aldrich) at 37°C, 5% CO_2_ [32]. Phleomycin (Invivogen) was used at 1 μg/ml, hygromycin (Sigma-Aldrich) at 1 μg/ml for bloodstream and 50 μg/ml for insect-stage, puromycin (Sigma-Aldrich) at 1 μg/ml, and blasticidin (Melford) at 5 μg/ml, as appropriate. RNAi was induced using tetracycline (Sigma-Aldrich) at 1 μg/ml. Differentiation into insect-stage cells was performed as described [59]. Briefly, 2 x 10^7^ cells were washed in DTM medium, and resuspended in 5 ml of DTM supplemented with 15% heat-inactivated fetal bovine serum, 3 mM *cis*-aconitate (Sigma-Aldrich) and 3 mM sodium isocitrate (Sigma-Aldrich). Cells were cultivated for at least 7 days at 27°C prior to analysis. Established insect-stage cells were cultured in SDM-79 medium (Gibco) supplemented with 10% heat-inactivated fetal bovine serum (Sigma-Aldrich), GlutaMAX (Gibco) and 2 mg/l hemin (Sigma-Aldrich) as described [60]. Genetic manipulation was performed by electroporation in cytomix using an Amaxa nucleofector (Lonza) for BSF cells, and a Gene Pulser (BioRad) for insect-stage cells.

### Plasmids and Cas9-based editing

A codon-optimised mNeonGreen (mNG) sequence was cloned using AvrII and PacI (NEB) restriction sites in the pNAT vector [61]. The *THT1/THT2* gene specific targets (nucleotides 4–359 for *THT1*, and 4–180 for *THT2*; GeneScript) were cloned using SmaI and XhoI (NEB) restriction sites. The resulting pNAT^mNG^THT1 was linearized with BaeI and pNAT^mNG^THT2 with BsrGI (NEB) prior to transfection. The pRPa^iSL^ vector [61] was used to assemble the RNAi constructs. The inserts comprised a *THT1*-specific sequence for pRPa^iSL^THT1 (nucleotides 145–392, 459–600, 704–793; GeneScript) and a common region targeting both *THT1* and *THT2*, for pRPa^iSL^THT1+2 (nucleotides 839–1338); these ‘stem-loop’ constructs were assembled using BamHI and XhoI (NEB) restriction sites. Cas9-based editing of Tb927.9.8720 was carried out using a previously described editing system [45]. Briefly, a Tb927.9.8720-specific sgRNA construct was assembled following annealing of the FbPgRNA5 and FbPgRNA3 oliogonucleotides. The resulting construct was linearised with NotI prior to transfection into 2T1^T7-Cas9^ cells. The NPT5^8720^ / NPT3^8720^ and PAC5^8720^ / PAC3^8720^ primer pairs were used to amplify repair-templates encoding the antibiotic selection markers and with terminal 25-bp Tb927.9.8720 untranslated region-specific targeting sequences. Cas9-based editing was induced for 24 h, at which point both repair templates (~5 μg of each) were transfected. Both antibiotics (G418 [Sigma-Aldrich] and puromycin [Sigma-Aldrich] at 2 μg/ml) were used to select for populations that lacked the Tb927.9.8720 gene, as demonstrated using a series of PCR-assays.

FbPgRNA5: AGGGAAGGTGCTCCCGCGCCTCTC

FbPgRNA3: AAACGAGAGGCGCGGGAGCACCTT

NPT5^8720^: TAACGACACCACTCTTCCCAGATTTCGGGTGCTCAAGCTGTGT

NPT3^8720^: CACACGCATCGAAGCAACCATTGGCGGGGAAGGAAACCAACTTG

PAC5^8720^: TAACGACACCACTCTTCCCAGATTTATGGGTCCCATTGTTTGCC

PAC3^8720^: CACACGCATCGAAGCAACCATTGGCACTATTTTCTTTGATGAAAGGG

### Western blot analysis

For western blot analysis, cells were harvested (1,000 g, 10 min), washed with 1 x PBS and lysed in Laemmli buffer (62 mM Tris pH 6.8, 10% glycerol, 2.3% SDS, 5% β-mercaptoethanol, bromphenol blue). To detect mNG-tagged THT proteins, samples were sonicated (3 cycles for 3s, 4°C), for VSG-2 and EP1 detection, samples were boiled at 95°C for 10 min. Equivalent of 10^7^ cells was loaded per well. Proteins were transferred from SDS gels onto Hybond ECL nitrocellulose membrane (GE Healthcare) using Trans-Blot Turbo Transfer System (BioRad) at 1.3 A, 25 V, for 10 min. Membranes were blocked in 5% milk in 0.005% PBS-Tween. Incubation with α-mNG antibody (Chromotek) was performed at 1:1,000, 4°C, overnight, α-VSG-2 at 1:10,000 for 1 h at room temperature (RT), α-EP1 (Cedarlane) at 1:1,000 for 1 h at RT, α-EF1 (Millipore) at 1:10,000 for 1 h at RT. Following three washes in PBS-Tween for 10 min, the secondary α-mouse or α-rabbit HRP-coupled antibody (BioRad) incubation was performed at 1:10,000, for 1 h at RT. Following a further three washes in PBS-Tween, the signal was visualised using an ECL kit (GE Healthcare) with a G:BOX chemidoc (Syngene).

### Microscopy and immunofluorescence microscopy assay

For microscopy, cells were washed with 1 x PBS and fixed in methanol-free 3% formaldehyde (Thermo Scientific) for 15 min at RT. Following two washes in PBS, cells were resuspended in 1% bovine serum albumin (Sigma-Aldrich) and allowed to dry on microscopy slides. For direct fluorescence microscopy, slides were immediately mounted with Vectashield with DAPI (Vector Laboratories). For immunofluorescence microscopy, slides were blocked with 50% FBS in PBS for 15 min at RT and, after two washes with PBS, primary α-VSG-2 (1:10,000; [62]) or α-EP1 (1:1,000; Cedarlane) antibodies were applied for 1 h at RT. Following 3 washes in PBS, the secondary antibodies, α-rat IgA-rhodamine (1:1,1000; Sigma-Aldrich) and α-mouse Alexa 568 (1:1,000; Life Technologies), respectively, were applied for 1 h at RT, followed by a further three washes in PBS. Images were captured using an Axiovert 200 epifluorescence microscope and processed using Zen imaging software (Zeiss).

### 2-^14^C(U)-deoxyglucose uptake assay

The uptake assay was performed as described previously [24] with minor modifications. Briefly, 10^8^ cells were harvested (1000 g, 10 min 4°C), washed twice in ice-cold transport buffer without glucose (33 mM HEPES, 98 mM NaCl, 4.6 mM KCl, 0.55 mM CaCl_2_, 0.07 mM MgSO_4_, 5.8 mM NaH_2_PO_4_, 0.3 mM MgCl_2_, 23 mM NaHCO_3_, pH 7.3) and resuspended to 10^8^ cells/ml in the same buffer. Uptake was initiated by adding 100 μl of cell suspension to 100 μl of transport buffer containing 0.25 μCi ^14^C-2-deoxyglucose (PerkinElmer) layered over 100 μl of dibutyl phthalate (Sigma-Aldrich). After incubation at 37°C or 4°C for the appropriate time, transport was stopped by centrifugation through the oil layer (16,000 g, 1 min). Microcentrifuge tubes were flash frozen in liquid nitrogen and the bottoms of the tubes, containing the cell pellets, were snipped into scintillation vials. Pellets were solubilised overnight in 150 μl of 1 M NaOH before mixing with 2 ml of scintillation fluid and radioactivity was measured on a scintillation counter (Beckman LS 6500) for 1 min.

### LC-MS metabolomic analysis

For the liquid chromatography–mass spectrometry (LC-MS) metabolomic analysis the sample extraction was performed as described previously [11]. THT1/THT2 knockdown cells were grown in the presence of tetracycline and 5 mM ^13^C_3_-U-glycerol (Sigma-Aldrich) three days prior to sample preparation. Briefly, 5 x 10^7^ cells were used for each final 200 μl sample. Cells were rapidly cooled in a dry ice/ethanol bath to 4°C, centrifuged at 1,300g, 4°C for 10 min, washed with 1 x PBS, and resuspended in extraction solvent (chloroform:methanol:water, 1:3:1 volume ratio). Following shaking for 1 h at 4°C, samples were centrifuged at 16,000g at 4°C for 10 min and the supernatant was collected and stored at -80°C. The analysis was performed using separation on 150 x 4.6 mm ZIC-pHILIC (Merck) on Dionex UltiMate 3000 RSLC (Thermo Scientific) followed by mass detection on an Orbitrap QExactive mass spectrometer (Thermo Fisher) at Glasgow Polyomics. Analysis was operated in polarity switching mode, using 10 μl injection volume and a flow rate of 300 μl/min. The samples were run alongside 170 authentic standards. The data were processed and analyzed using mzMatch software [63] and mzMatchISO [64]. The analysis was performed in 4 replicates, means of which are indicated, non-labelled samples were run in parallel. Metabolites were identified based on matches with standards or were predicted based on mass and retention time. Metabolomics data have been deposited to the EMBL-EBI MetaboLights database (https://www.ebi.ac.uk/metabolights/index) with the identifier MTBLS706.

### Fructose-1,6-bisphosphatase activity assay

To measure FbPase activity, cells were harvested, washed in PBS, and resuspended in TE buffer (10 mM Tris-HCl pH 8.0, 1 mM EDTA, 0.15% Triton X-100, cOmplete Protease Inhibitor Cocktail [Roche]) at 2 x 10^8^ cells/ml. Following 20 min incubation at RT, cell extracts were centrifuged at 14,000 g, 16°C, 10 min, and supernatants were collected and kept on ice. The reaction mixture (20 mM Tris pH 7.8, 10 mM MgCl_2_, 1 mM NADP, 1 μl glucose-6-phosphate isomerase [Sigma-Aldrich], 1 μl glucose-6-phosphate dehydrogenase [Sigma-Aldrich], 100 μl cell extract in H_2_O) was incubated at 30°C for 5 min and 5 mM fructose 1,6-bisphosphate was added immediately prior to reading at 340 nm, 30°C with an UV-1601 spectrophotometer (Shimadzu).

### VSG glycosylation analysis

The VSG isolation was performed as described previously with minor modifications [65]. The cells were cultured in 5 mM ^13^C_3_-U-glycerol (Sigma-Aldrich) for three days prior to sample preparation, and unlabelled samples were prepared alongside. 10^8^ cells were harvested (1,300 g, 4°C, 10 min), washed twice in ice-cold PBS and resuspended in 300 μl of 10 mM Na_2_HPO_4_ pH 8.0 in MS grade water, containing 0.1 mM TLCK (Sigma-Aldrich), 1 μg/ml leupeptin (Sigma-Aldrich), 1 μg/ml apoprotinin (Sigma-Aldrich), and 10 mM PMSF (Sigma-Aldrich). Following 5 min incubation at 37°C, the samples were cooled on ice and centrifuged at 14,000 g, 4°C for 5 min. The samples were applied on chromatography columns containing 400 μl of bead mixture (50:50 volume ratio, Anion Exchange Cellulose DE52 (Whatman) in 10 mM Na_2_HPO_4_ pH 8.0 buffer) and eluted with 800 μl of 10 mM Na_2_HPO_4_ pH 8.0. The obtained eluate was concentrated and diafiltered against water using Millipore Amicon Ultra-0.5 Centrifugal Filter Devices (Merck) following the manufacturer’s instructions. Carbohydrate compositional analysis was performed by GC-MS. Samples (6–10 μg) were mixed with 2 nmol *scyllo*-inositol internal standard and dried using vacuum centrifugation. Dried samples were then subjected to methanolysis by adding 50 μl of 0.5 M HCl in dry methanol and incubating at 85°C for 4 h. Methanolysates were re-N-acetylated by the addition of 10 μl pyridine and 10 μl acetic anhydride and incubating at RT for 30 min. The samples were dried under vacuum and derivatised with 15 μl trimethysilylation (TMS) reagent at RT for 30 min. 1 μl aliquots of each sample was injected in GC-MS (Agilent Technologies, 7890B Gas Chromatography system with 5977A MSD) equipped with Agilent J&W HP-5ms GC Column (30 m X 0.25 mm, 0.25 μm) with He carrier gas at 0.5 ml/min. The temperature program used was run over 32.5 min with 95°C (for 1 min) - 140°C (30°C/min) to 265°C at 5°C/min (for 5 min). The mass spectra were collected from linear scanning over *m/z* 50–650, and quantification was based on the integration of the extracted ion-current chromatograms and empirically determined molar response factors.

## Supporting information

S1 Fig THT knockdown in bloodstream and insect stage cells. (A) The protein blot shows native tagged ^mNG^THT2 in bloodstream form cells following THT1 knockdown for 5 days. (B) The protein blot shows depletion of native tagged ^mNG^THT2 following THT1/THT2 knockdown in insect stage cells; see Fig 2C for depletion of ^mNG^THT1 by the same approach in bloodstream form cells. (C) Bloodstream form THT1/THT2 knockdown cells were grown in the presence of tetracycline and glycerol for up to 6 days, and scrutinised by immunofluorescence microscopy. Staining of the cell surface with α-VSG-2, but not α-EP procyclin antibody validated that these cells are not differentiated into PCF. A PCF cell is shown as a control. DNA was counter stained with DAPI; scale bars 5 μm.
(PDF)

S2 Fig Additional metabolites detected by the LC-MS analysis. The size of the bars represents the total abundance, and coloured parts indicate ^13^C labelling as depicted in the legend. The samples are from WT cells, WT grown in ^13^C-glycerol, THT1/THT2 RNAi grown in ^12^C-glycerol and THT1/THT2 RNAi grown in ^13^C-glycerol. Natural abundance of ^13^C is 1%, hence the 1C labelling in ‘un-labelled’ samples.
* the identity of these metabolites was confirmed using a match with a standard.
(PDF)

S3 Fig Measurement of incorporation of ^13^C glycerol into the mannose and galactose residues of VSG. Total ion chromatograms of the methyl-glycoside TMS derivatives from wild-type (panels A-B) and THT1/THT2 knockdown trypanosomes (panels C-D) grown in the absence and presence of ^13^C glycerol, respectively. The peaks due to mannose (Man), galactose (Gal) and the *scyllo*-inositol internal standard (s-I) are indicated. The insets show a detail of the electron impact mass spectra of the main Man peak, illustrating the natural abundance of m/z 206 relative to m/z 204 (panel A) compared to a sample where ^13^C has been incorporated into the VSG sugar residues (panel D). One representative replicate, n = 3.
(PDF)

S1 Table LC-MS metabolomics data with ^13^C_3_-glycerol. The samples are from WT cells, WT grown in ^13^C-glycerol, THT1/THT2 RNAi grown in ^12^C-glycerol and THT1/THT2 RNAi grown in ^13^C-glycerol, n = 4.
(XLSX)
