# Supplementary material for: Gluconeogenesis using glycerol as a substrate in bloodstream-form Trypanosoma brucei
Source: PLoS Pathog. 2018 Dec 27;14(12):e1007475. doi: 10.1371/journal.ppat.1007475 (PMC6307712; doi:10.1371/journal.ppat.1007475)
Supplement: S1 Fig — (A) The protein blot shows native tagged mNGTHT2 in bloodstream form cells following THT1 knockdown for 5 days. (B) The protein blot shows depletion of native tagged mNGTHT2 following THT1/THT2 knockdown in insect stage cells; see Fig 2C for depletion of mNGTHT1 by the same approach in bloodstream form cells. (C) Bloodstream form THT1/THT2 knockdown cells were grown in the presence of tetracycline and glycerol for up to 6 days, and scrutinised by immunofluorescence microscopy. Staining of the cell surface with α-VSG-2, but not α-EP procyclin antibody validated that these cells are not differentiated into PCF. A PCF cell is shown as a control. DNA was counter stained with DAPI; scale bars 5 μm. (PDF) [file ppat.1007475.s001.pdf]

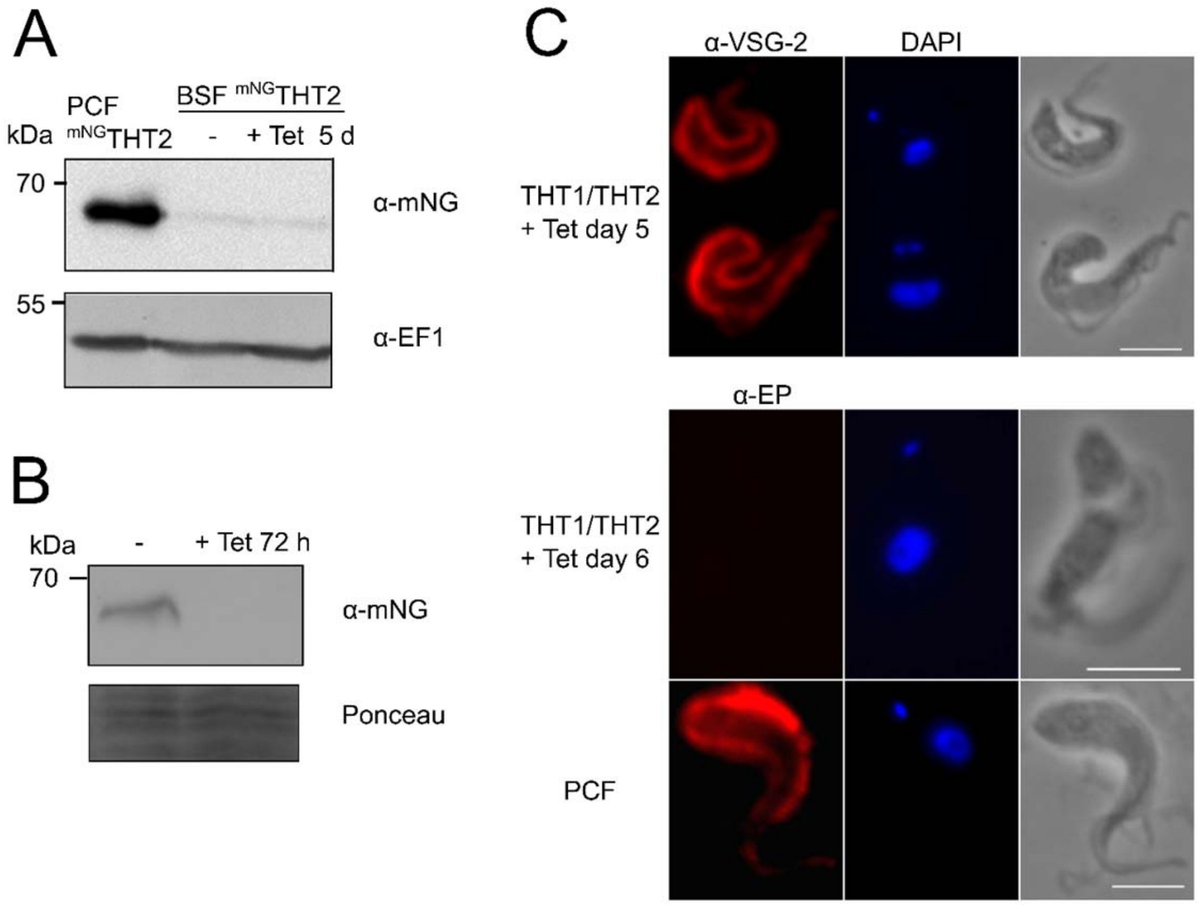

S1 Fig. THT knockdown in bloodstream and insect stage cells. (A) The protein blot shows native tagged ^{mNG}THT2 in bloodstream form cells following THT1 knockdown for 5 days. (B) The protein blot shows depletion of native tagged ^{mNG}THT2 following THT1/THT2 knockdown in insect stage cells; see Fig 2C for depletion of ^{mNG}THT1 by the same approach in bloodstream form cells. (C) Bloodstream form THT1/THT2 knockdown cells were grown in the presence of tetracycline and glycerol for up to 6 days, and scrutinised by immunofluorescence microscopy. Staining of the cell surface with α-VSG-2, but not α-EP procyclin antibody validated that these cells are not differentiated into PCF. A PCF cell is shown as a control. DNA was counter stained with DAPI; scale bars 5 μm.
