# Supplementary material for: Gluconeogenesis using glycerol as a substrate in bloodstream-form Trypanosoma brucei
Source: PLoS Pathog. 2018 Dec 27;14(12):e1007475. doi: 10.1371/journal.ppat.1007475 (PMC6307712; doi:10.1371/journal.ppat.1007475)
Supplement: S2 Fig — The size of the bars represents the total abundance, and coloured parts indicate 13C labelling as depicted in the legend. The samples are from WT cells, WT grown in 13C-glycerol, THT1/THT2 RNAi grown in 12C-glycerol and THT1/THT2 RNAi grown in 13C-glycerol. Natural abundance of 13C is 1%, hence the 1C labelling in ‘un-labelled’ samples. * the identity of these metabolites was confirmed using a match with a standard. (PDF) [file ppat.1007475.s002.pdf]

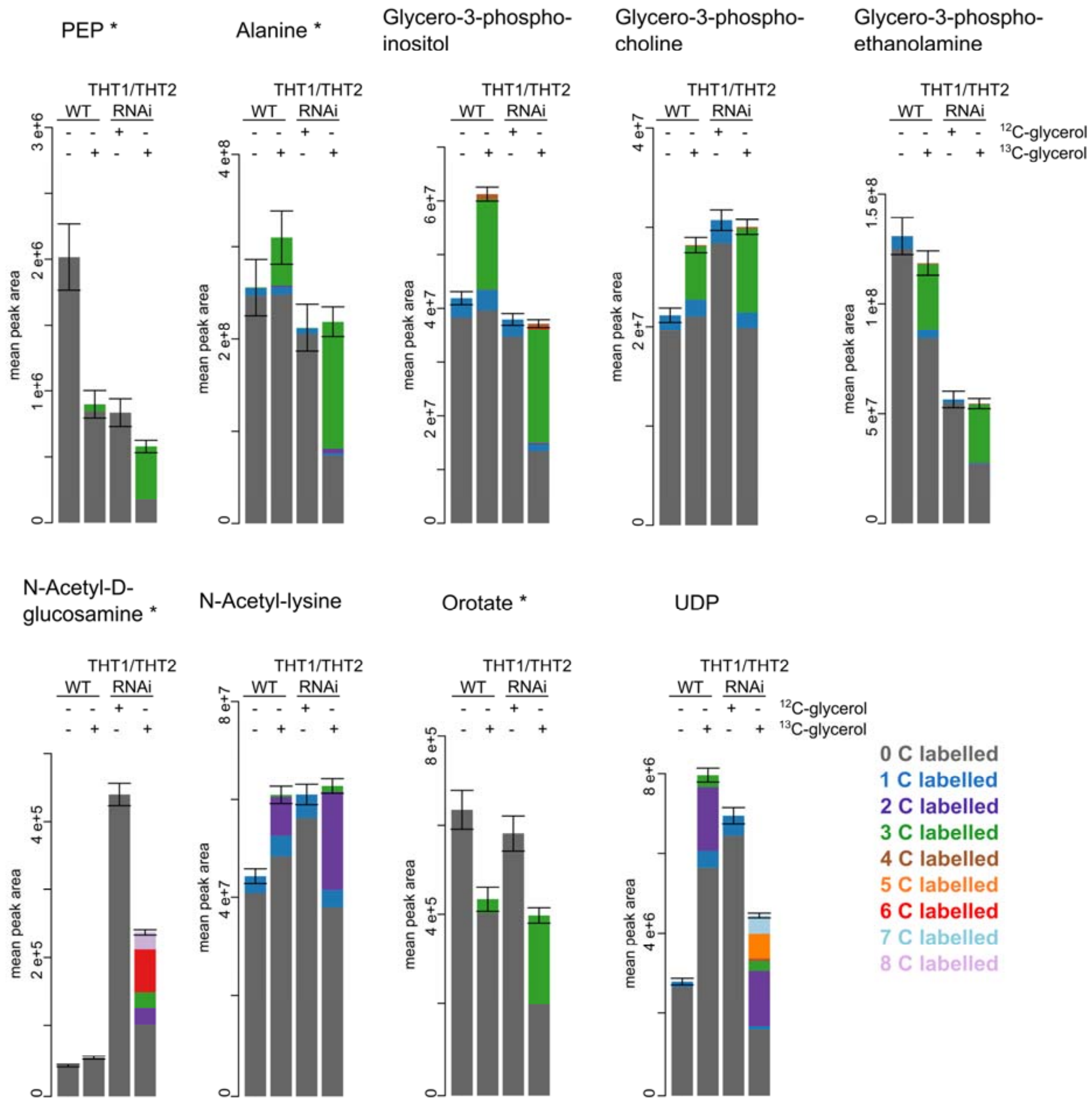

S2 Fig. Additional metabolites detected by the LC-MS analysis. The size of the bars represents the total abundance, and coloured parts indicate ^{13}C labelling as depicted in the legend. The samples are from WT cells, WT grown in ^{13}C-glycerol, THT1/THT2 RNAi grown in ^{12}C-glycerol and THT1/THT2 RNAi grown in ^{13}C-glycerol. Natural abundance of ^{13}C is 1 %, hence the labelling in ‘un-labelled’ samples.

* the identity of these metabolites was confirmed using a match with a standard.
