# Supplementary material for: Gluconeogenesis using glycerol as a substrate in bloodstream-form Trypanosoma brucei
Source: PLoS Pathog. 2018 Dec 27;14(12):e1007475. doi: 10.1371/journal.ppat.1007475 (PMC6307712; doi:10.1371/journal.ppat.1007475)
Supplement: S3 Fig — Total ion chromatograms of the methyl-glycoside TMS derivatives from wild-type (panels A-B) and THT1/THT2 knockdown trypanosomes (panels C-D) grown in the absence and presence of 13C glycerol, respectively. The peaks due to mannose (Man), galactose (Gal) and the scyllo-inositol internal standard (s-I) are indicated. The insets show a detail of the electron impact mass spectra of the main Man peak, illustrating the natural abundance of m/z 206 relative to m/z 204 (panel A) compared to a sample where 13C has been incorporated into the VSG sugar residues (panel D). One representative replicate, n = 3. (PDF) [file ppat.1007475.s003.pdf]

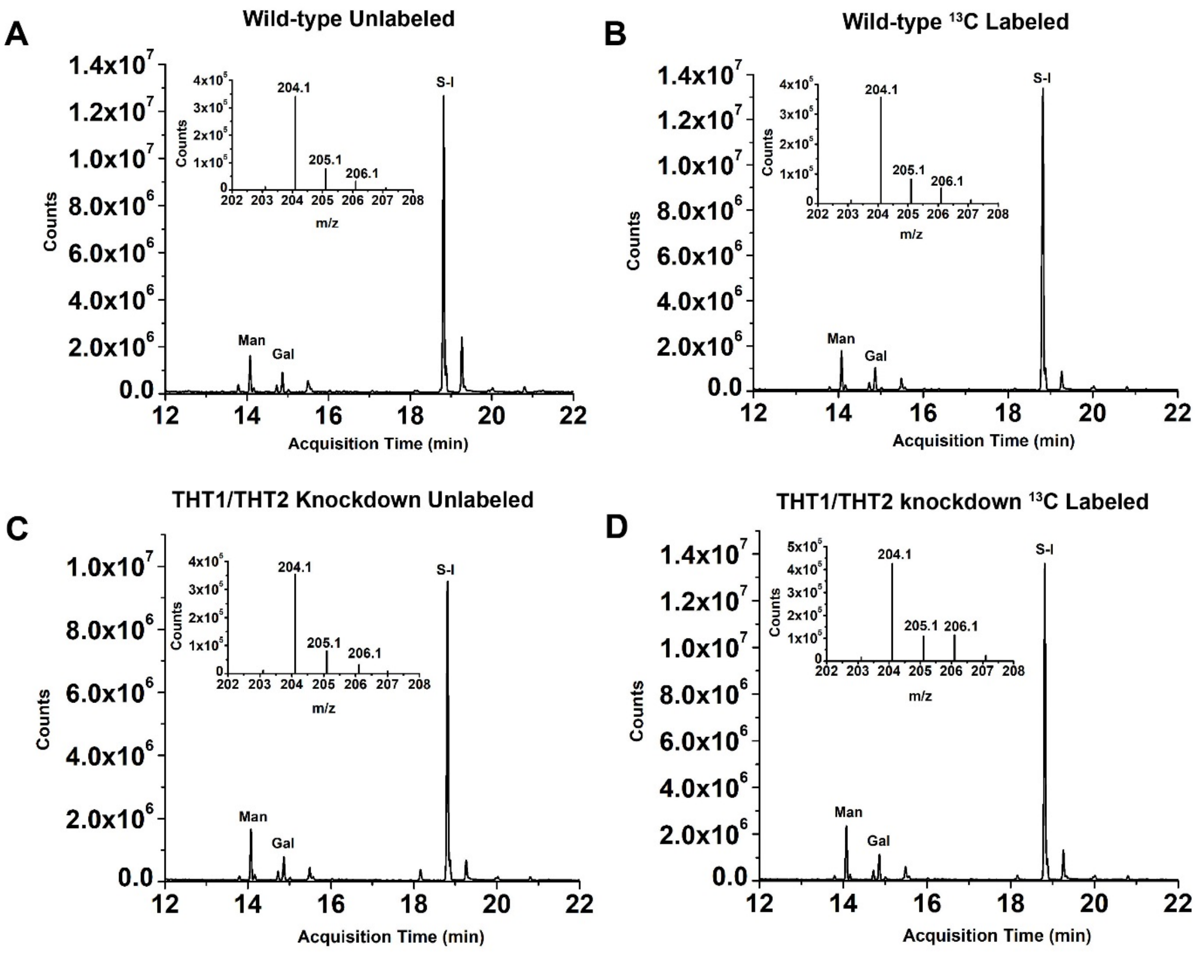

S3 Fig. Measurement of incorporation of ^{13}C glycerol into the mannose and galactose residues of VSG. Total ion chromatograms of the methyl-glycoside TMS derivatives from wild-type (panels A-B) and THT1/THT2 knockdown trypanosomes (panels C-D) grown in the absence and presence of ^{13}C glycerol, respectively. The peaks due to mannose (Man), galactose (Gal) and the *scyllo*-inositol internal standard (s-I) are indicated. The insets show a detail of the electron impact mass spectra of the main Man peak, illustrating the natural abundance of m/z 206 relative to m/z 204 (panel A) compared to a sample where ^{13}C has been incorporated into the VSG sugar residues (panel D). One representative replicate, n = 3.
